# Supplementary material for: Kinase-dead ATR differs from ATR loss by limiting the dynamic exchange of ATR and RPA
Source: Nat Commun. 2018 Dec 17;9:5351. doi: 10.1038/s41467-018-07798-3 (PMC6297235; doi:10.1038/s41467-018-07798-3)
Supplement: Supplementary file 1 — Supplementary Information file [file 41467_2018_7798_MOESM1_ESM.pdf]

Kinase-dead ATR differs from ATR loss by limiting the dynamic exchange of ATR and RPA

Menolfi et al.

Supplementary Figure 1

a

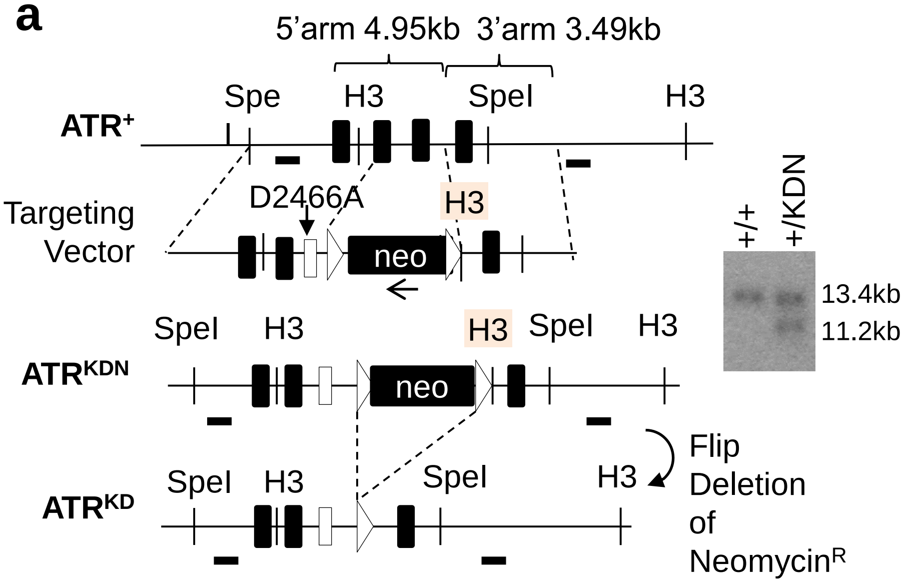

b

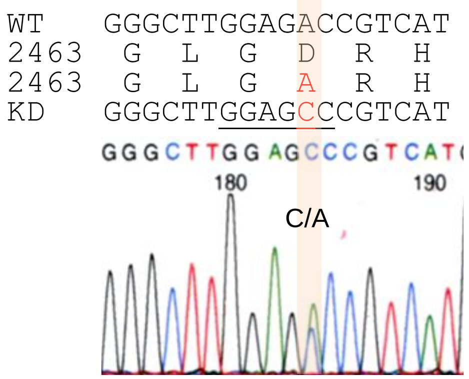

c

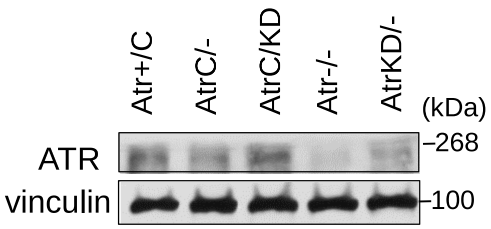

d

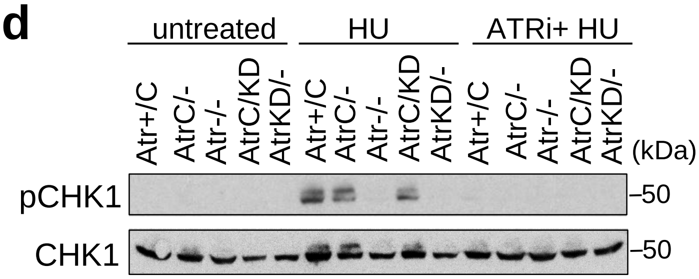

e

Genotype	Expected frequency	Observed mice	Observed frequency
Atr+/+	50%	38	48.10%
Atr+/KD	50%	41	51.90%

g

Genotype	Observed mice	Observed frequency	Fisher's exact test
Atr+/+	18	28.12%	p=0.8416
Atr+/-	22	34.38%	p=0.3335
Atr+/KD	24	37.50%	p=0.1816
AtrKD/-	0	0%	**p<0.0001**
tot	64	100%	

f

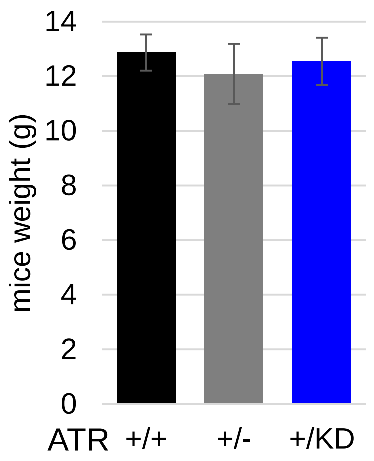

h

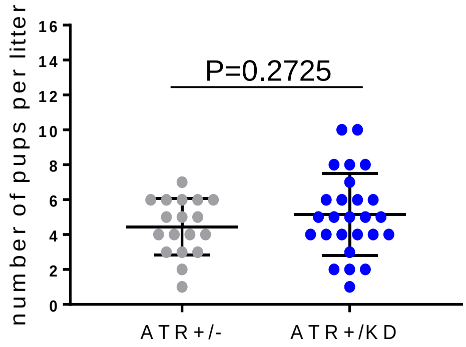

Supplementary Figure 1 Generation and validation of ATR kinase dead (KD) mutation (**a**) Strategy for the generation of the *Atr-KD* allele. Southern blot confirmed the insertion of neomycin gene in embryonic stem (ES) cells. DNA was digested with HindIII and a 3' probe was used in order to detect the WT allele (13.4 kb) and the targeted allele (11.2 kb). (**b**) The expression of the mutated D2466A allele was confirmed by sequencing of reverse transcribed cDNA extracted from $Atr^{+/KD}$ cells. (**c**) $Atr^{+/C}$, $Atr^{C/-}$, $Atr^{C/KD}$, $Atr^{-/-}$ and $Atr^{KD/-}$ ($Atr^{C/-}$ and $Atr^{C/KD}$ MEFs treated with 500nM of 4OH-tamoxifen for 96 hours respectively) derived whole-cell extracts were immunoblotted for ATR protein and vinculin. (**d**) $Atr^{+/C}$, $Atr^{C/-}$, $Atr^{C/KD}$, $Atr^{-/-}$ and $Atr^{KD/-}$ ($Atr^{C/-}$ and $Atr^{C/KD}$ treated with 500nM of 4OH-tamoxifen for 96 hours respectively) MEFs were left untreated or treated with 0.2 mM HU for 1 hour with or without 10 µM ATRi (VE-821). Whole-cell extracts (WCE) were immunoblotted with CHK1 Ser245 and total CHK1 antibodies. (**e**) Breedings between $Atr^{+/+}$ males and $Atr^{+/KD}$ females were established. Table reports the number and the percentages of $Atr^{+/+}$ and $Atr^{+/KD}$ mice obtained from several breedings. (**f**) The weight (gram) of 3 weeks old female mice of different genotypes. The bars represent the means ± SD of the weight of eight $Atr^{+/+}$, eight $Atr^{+/-}$ and nine $Atr^{+/KD}$ mice. The p value is > 0.15 between each pair using t test. (**g**) Breedings between $Atr^{+/-}$ males and $Atr^{+/KD}$ females were established. Table reports the number and the percentages of $Atr^{+/+}$, $Atr^{+/-}$, $Atr^{+/KD}$ and $Atr^{KD/-}$ mice observed from several breedings. Fisher's exact test was used to calculate the p values of significance. (**h**) The number of pups from $Atr^{+/-}$ and $Atr^{+/KD}$ females, in breedings with $Atr^{+/+}$ males, were counted from several littermates and unpaired two-tailed t test was used for the statistical analysis.

Source data are provided as a Source Data file.

Supplementary Figure 2

a

		Frequency of TUNEL+ cells per tubule		
Genotype	Tubules counted	0	1-2	>3
ATR+/+	160	97%	3%	0%
ATR+/-	121	91%	7%	2%
ATR+/KD	41	29%	54%	17%
ATR+/KD	17	64%	18%	18%

b

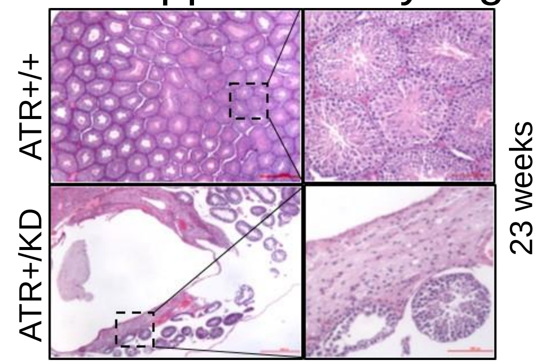

c

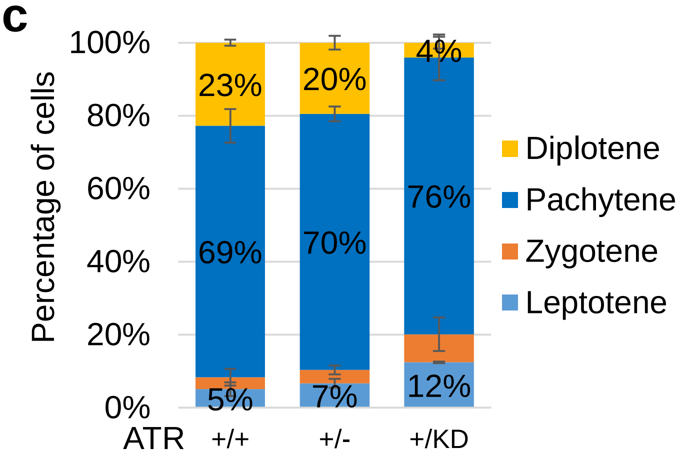

d

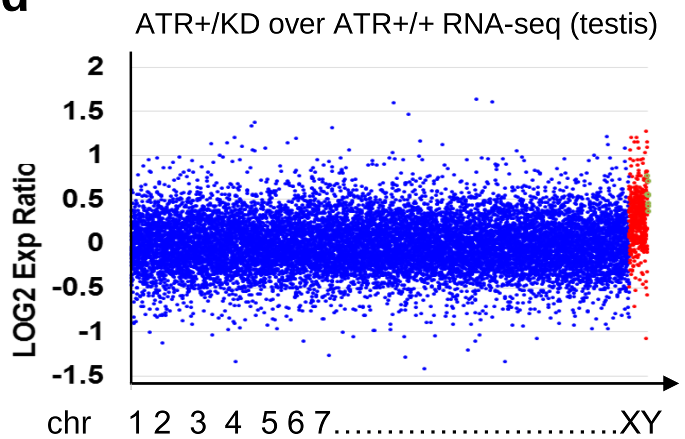

e

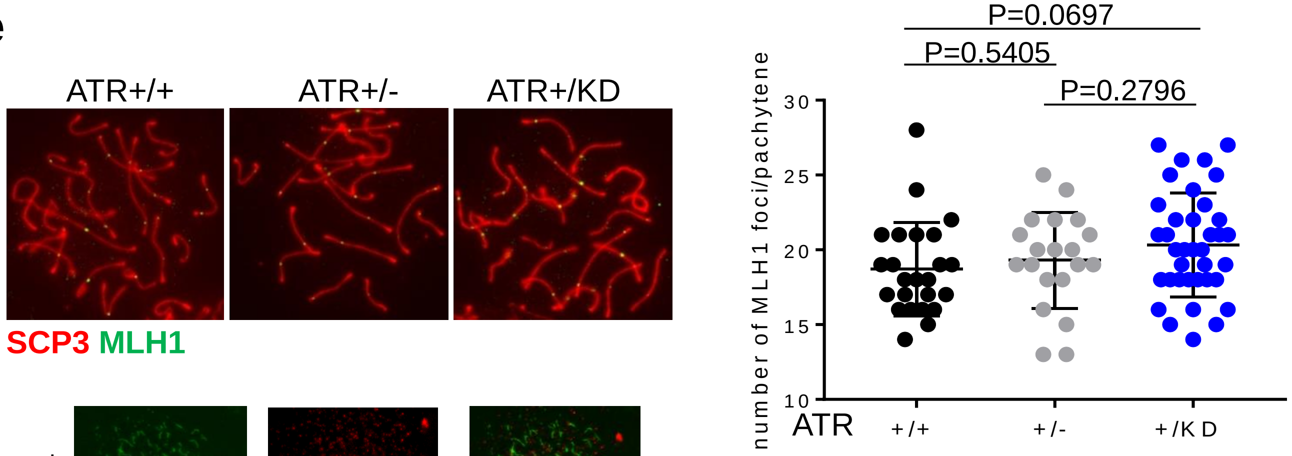

f

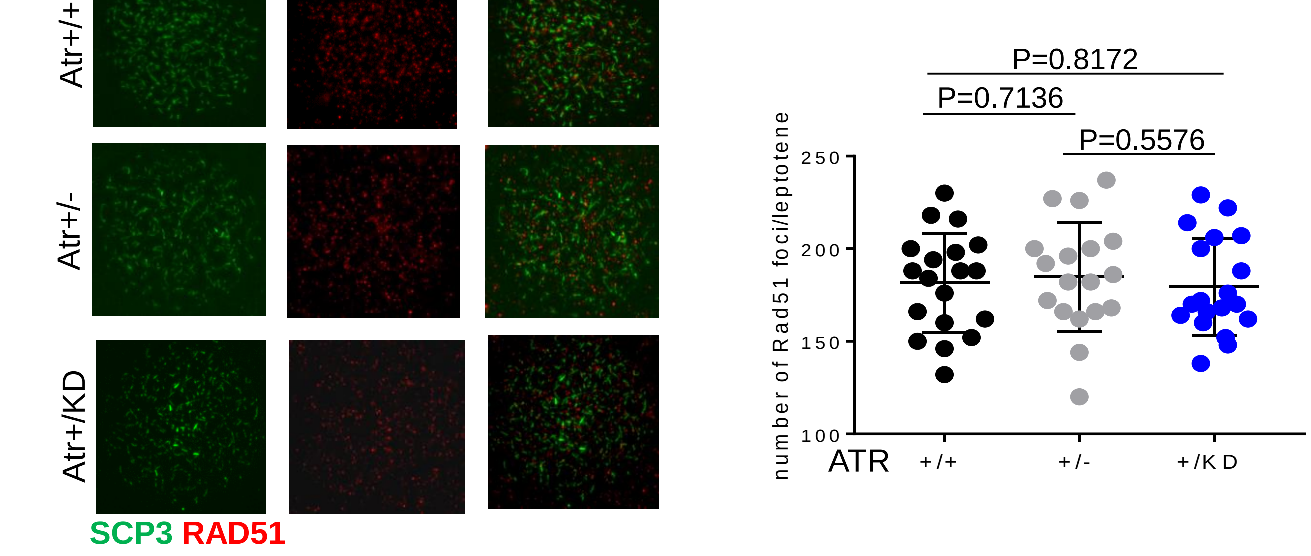

Supplementary Figure 2. Meiosis phenotypes of *Atr*$^{+/KD}$ male mice. (**a**) Table reports the analysis of TUNEL positive cells performed on testes form 15 weeks old *Atr*$^{+/+}$, *Atr*$^{+/-}$ and *Atr*$^{+/KD}$ mice. The total number of tubules counted per each genotype and the frequency of tubules with 0, 1-2 or >3 TUNEL positive cells are reported. (**b**) Histological sections and zoomed images of 23 weeks old *Atr*$^{+/+}$ and *Atr*$^{+/KD}$ mice. *Atr*$^{+/KD}$ mice show atrophic testes with enlarged and severely cystic architecture, while *Atr*$^{+/+}$ mice have testes with well-organized seminiferous tubules. (**c)** The percentages of cells in different stages of prophase I (leptotene, zygotene, pachytene and diplotene) were analyzed from *Atr*$^{+/+}$, *Atr*$^{+/-}$ and *Atr*$^{+/KD}$ mice (3 mice per genotype). *Atr*$^{+/KD}$ testis showed a significant increase in cells in leptotene (*Atr*$^{+/+}$ vs *Atr*$^{+/KD}$ P=0.0169, *Atr*$^{+/-}$ vs *Atr*$^{+/KD}$ P=0.0109) and a significant decrease in cells in diplotene (*Atr*$^{+/+}$ vs *Atr*$^{+/KD}$ P=0.0005, *Atr*$^{+/-}$ vs *Atr*$^{+/KD}$ P=0.0035). Unpaired two-tailed t test was used (**d**) RNA-sequencing from 7 weeks old *Atr*$^{+/+}$and *Atr*$^{+/KD}$ testes. Overexpression of X-Y genes in *Atr*$^{+/KD}$ testes, compared to *Atr*$^{+/+}$ testes, is represented in red. (**e**) Spermatocytes spreads were stained with anti-SCP3 and anti-MLH1 antibodies. Representative pachytenes for each genotype are shown. The number of MLH1 foci per pachytene was counted and unpaired two-tailed t test was used. (**f**) Spermatocytes spreads were stained with anti-SCP3 and anti-RAD51 antibodies. Representative images of leptotene cells are shown. The number of Rad51 foci per leptotene was counted using Cell Counter (ImageJ) and unpaired two-tailed t test was used.

Source data are provided as a Source Data file.

Supplementary Figure 3

a

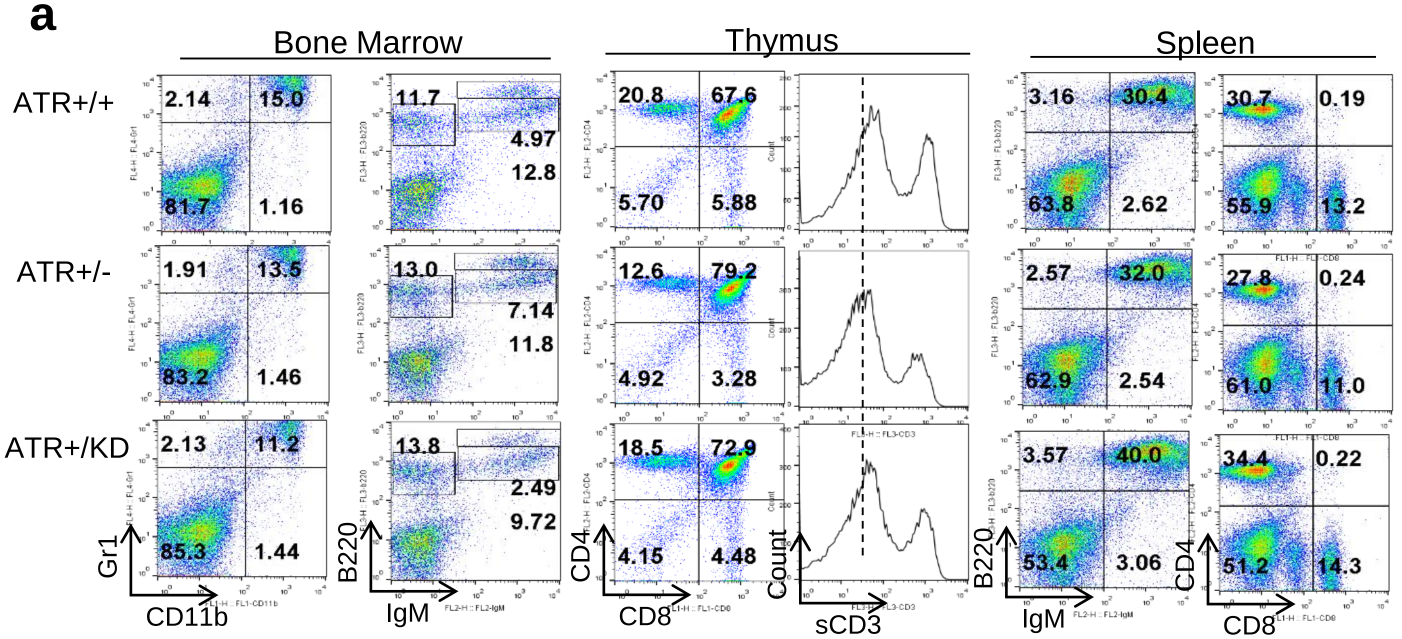

b

(X 10^6)	Thymus					Spleen		
	Total	DN	DP	CD4+	CD8+	Total	CD4+	CD8+
Atr+/+(n=3)	75.5±4.1	4.2±0.9	52.4±3.8	15.4±5.5	3.6±1.2	53.3±4.8	14.7±1.5	5.9±0.4
Atr+/-(n=3)	76.6±3.2	5.8±1.7	52.5±7.5	14.8±5.6	3.5±1.1	45.3±6.8	14.2±3.8	5.5±1.2
Atr+/KD(n=4)	47.3±11.2	2.0±0.5	35.9±7.8	7.5±2.6	1.9±0.7	44.0±12.6	11.8±2.5	4.5±1.1

	Bone Marrow							Spleen (X 10^6)	
	Lymph* (%)	Pro-B(%)	Pre-B(%)	IgM+(%)	Pre/Pro	IgM+/Pre	IgM+ B220$^{H/Mid}$	Total	B220+IgM+
Atr+/+(n=3)	21.1±3.7	7.5±0.7	9.5±2.3	15.9±3.8	1.25±0.21	1.77±0.66	0.44±0.07	53.3±4.8	14.0±4.6
Atr+/-(n=3)	23.5±2.5	7.2±0.6	9.5±4.1	16.8±2.0	1.29±0.47	2.08±0.97	0.49±0.05	45.3±6.8	11.2±4.5
Atr+/KD(n=4)	20.6±2.3	6.9±1.3	6.9±2.2	16.9±3.1	1.00±0.26	2.70±1.11	0.27±0.03	44.0±12.6	11.6±1.6

c

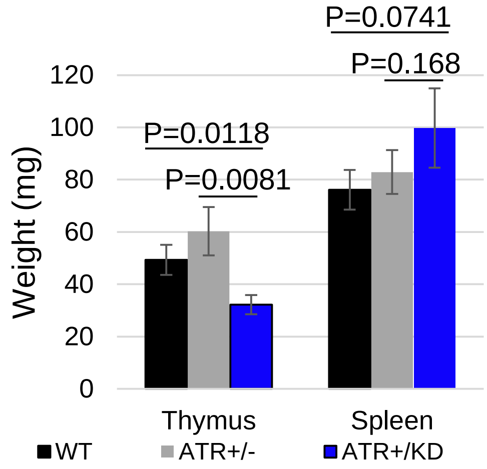

d

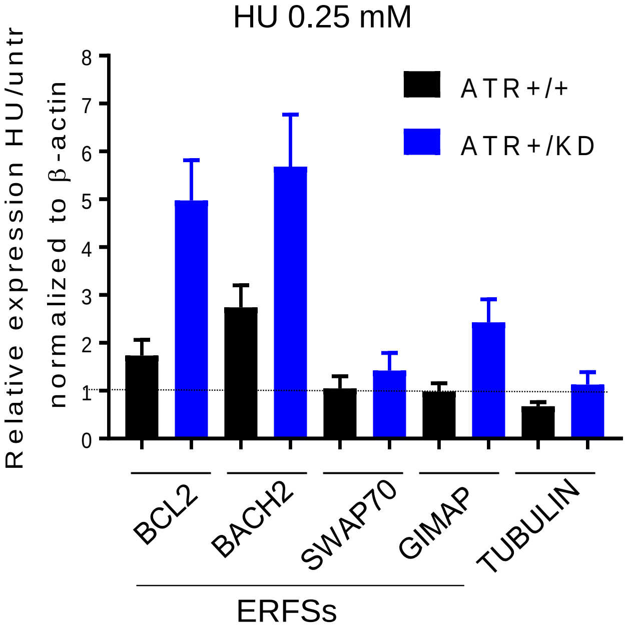

Supplementary Figure 3 Lymphocyte development in *Atr*$^{+/KD}$ mice**.** (**a**) Representative flow cytometry analyses of bone marrow myeloid (Cd11+ Gr1+) and B cells (IgM+ B220+), thymic CD4+CD8+ and CD3+ cells, splenic B (IgM^{+}B220^{+}) and T (CD4^{+}CD8^{+}) cells from 7-8 weeks old *Atr*$^{+/+}$, *Atr*$^{+/-}$ and *Atr*$^{+/KD}$ mice. (**b**) Tables report raw values for thymic, splenic and bone marrow cell populations from several *Atr*$^{+/+}$, *Atr*$^{+/-}$ and *Atr*$^{+/KD}$ mice. DN= double negative; DP= double positive (CD8+ CD4+). (**c**) The weights in mg of thymuses and spleens from several *Atr*$^{+/+}$, *Atr*$^{+/-}$ and *Atr*$^{+/KD}$ were plotted. The bar represent the means ± SD and unpaired two-tailed t test was used. (**d**) *Atr*$^{+/+}$ and *Atr*$^{+/KD}$ in vitro activated B cells were treated with 0.25 mM HU and RNA was collected for analysis of Early Replicating Fragile Sites (ERFSs) expression. Tubulin and ERFSs (*BCL2*, *BACH2*, *SWAP70*, *GIMAP*) were analyzed by RT-PCR and the ratio between HU and untreated, each normalized with β-actin, was plotted. Error bars represent the mean ± SD derived from biological triplicates.

Source data are provided as a Source Data file.

Supplementary Figure 4

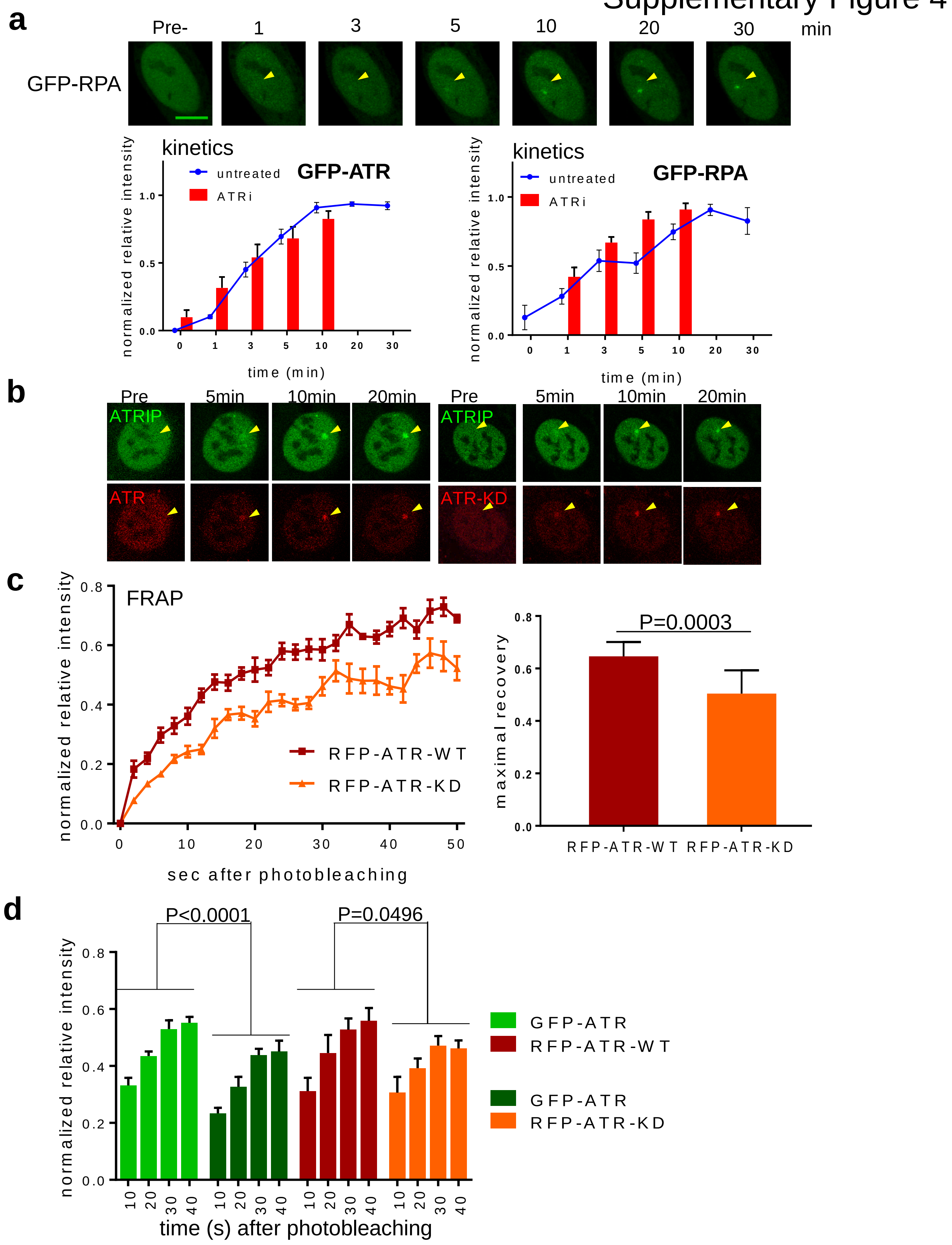

Supplementary Figure 4 The rapid exchange of ATR on damaged chromatin is dependent on its kinase activity**.** (**a**) The kinetics of GFP-ATR and GFP-RPA recruitment to sites of DNA damage. U2OS cells stably expressing GFP-ATR or transiently expressing GFP-RPA were cultured in the presence or absence of ATR inhibitor (ATRi, VE-821) and then monitored by confocal microscopy at the indicated time points after micro-irradiation (405 nm). Images of a representative GFP-RPA expressing cell are shown above (scale bar is 10 μm) and the relative intensities of GFP-ATR and GFP-RPA foci are plotted below as the mean ± SEM. (**b**) Representative images of U2OS cells transfected with RFP-ATR WT or RFP-ATR-KD (both with GFP-ATRIP) show that ATR-WT and ATR-KD can both be efficiently recruited to the site of laser damage. The yellow arrows point to the site of laser damage. (**c**) Recovering after photo bleaching for RFP-ATR-KD (orange line) and RFP-ATR-WT (red line) at a function of time (0-50 seconds) after photo bleaching. The photo bleach was performed 10 min after initial damage. The relative recovery at each time point is shown as the mean and SEM of all the cells collected. The bar graph on the right shows the mean and SEM of the maximal recovery. The p value was derived from unpaired two-tailed t test. (**d**) FRAP experiments performed on U2OS cells with stable expression of GFP-ATR (WT), which were then transfected with either RFP-ATR-WT or RFP-ATR-KD (both with FLAG-ATRIP). The graph shows the normalized relative intensity as a fraction of pre-bleached intensity at 10, 20, 30 and 40 seconds after photo bleaching. In cells expressing RFP-ATR-KD, the recovery of both GFP-ATR-WT and RFP-ATR-KD are similar and are both significantly less efficient than those in cells expressing GFP and RPF tagged ATR-WT at the same time. Linear regression model with degrees of freedom was used to calculate the p value between each sets (see methods for details).

Source data are provided as a Source Data file.

Supplementary Figure 5

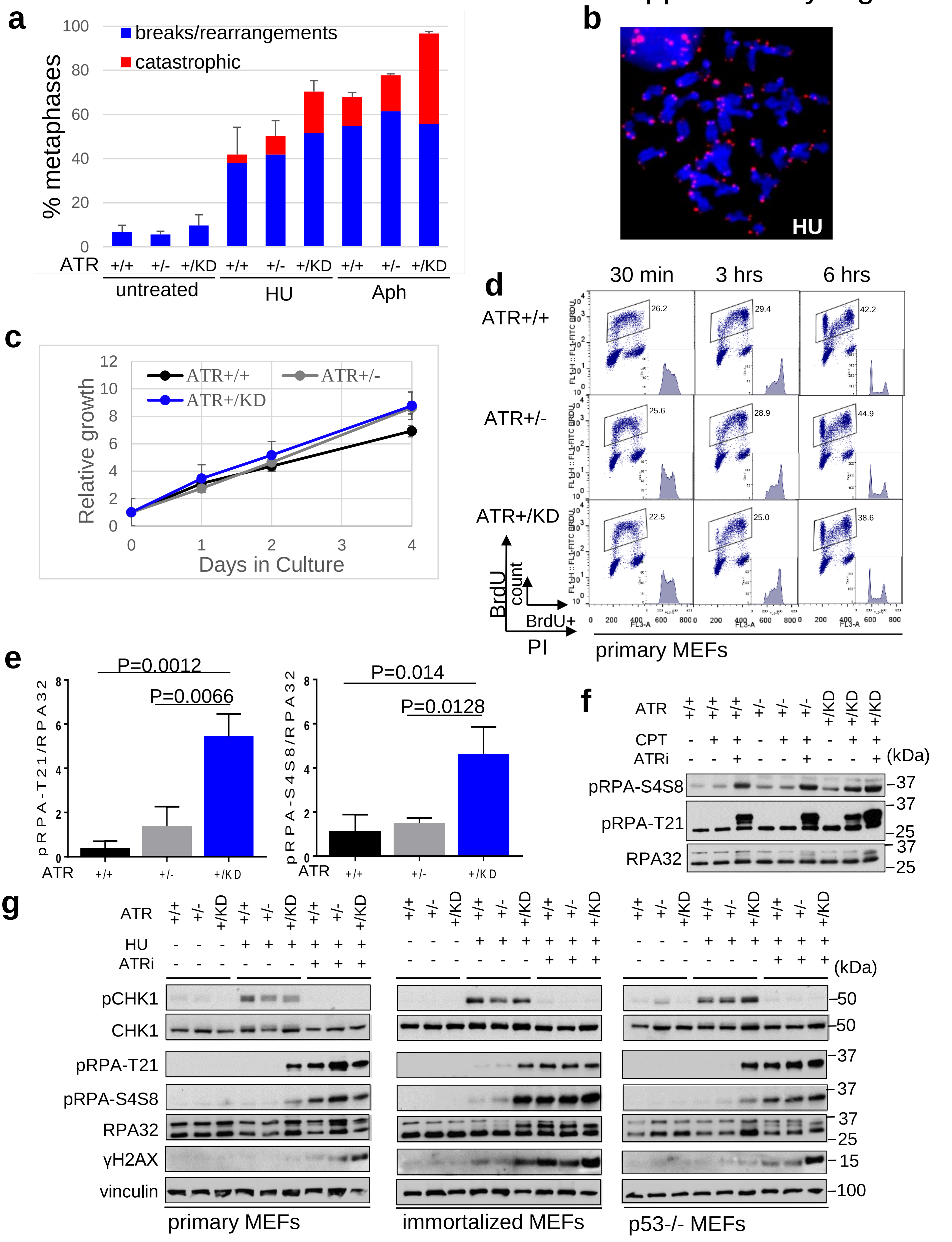

Supplementary Figure 5 Checkpoint responses in *Atr*$^{+/KD}$ cells (**a**) Metaphases from untreated, HU (0.25 mM) or Aphidicolin (0.5 µM) were divided in two categories: with breaks and complex rearrangements (fusions, quadri-radials) or catastrophic (>15 aberrations per metaphase). Fragile telomeres were not included. Three (HU) or two (untreated, Aphidicolin) independent experiments were performed. Unpaired two-tailed t test was used. In untreated conditions the p values between the different pairs are not significant. In HU, despite the qualitative increase in genome instability in *Atr*$^{+/KD}$ cells, p values do not reach the statistical significance (*Atr*$^{+/+}$ vs *Atr*$^{+/-}$ P=0.5783, *Atr*$^{+/+}$ vs *Atr*$^{+/KD}$ P=0.0968, *Atr*$^{+/-}$ vs *Atr*$^{+/KD}$ P=0.0753). In Aphidicolin, *Atr*$^{+/-}$ cells display significant increase in genome instability compared to *Atr*$^{+/+}$ cells (P=0.0448), while *Atr*$^{+/KD}$ cells display a significant increase in genome instability compared to both *Atr*$^{+/+}$ cells (P=0.0059) and *Atr*$^{+/-}$ cells (P=0.0042). (**b**) Representative catastrophic mitosis from *Atr*$^{+/KD}$ B cells treated with HU. (**c**) Primary *Atr*$^{+/+}$, *Atr*$^{+/-}$ and *Atr*$^{+/KD}$ MEFs were plated in 96-well plates and cell growth was monitored with the CyQuant DNA stain up to 4 days. Cell survival is reported relative to day 1 and the data represent the mean ± SD of three replicates per condition. (**d**) Primary *Atr*$^{+/+}$, *Atr*$^{+/-}$ and *Atr*$^{+/KD}$ MEFs were pulse-labeled with BrdU for 30 min, washed and released in unchallenged media up to 6 hours. Dot plots show the percentage of BrdU-positive cells and histograms show the cell cycle distribution of BrdU-positive cells. (**e**) Histograms show the quantification of pRPA-T21/RPA32 and pRPA-S4S8/RPA32 at 0.2 mM HU derived from three independent experiments (see Fig. 4f). The means ± SD are shown and unpaired two-tailed t test was used. (**f**) *Atr*$^{+/+}$, *Atr*$^{+/-}$ and *Atr*$^{+/KD}$ MEFs were treated with 200nM CPT for 2 hours, pre-treated or not with 10µM of ATRi (VE-821) for 1 hour. Whole-cell extracts (WCE) were immunoblotted with the indicated antibodies. (**g**) Primary, immortalized and *p53*$^{-/-}$ *Atr*$^{+/+}$, *Atr*$^{+/-}$ and *Atr*$^{+/KD}$ MEFs were treated with 0.2 mM HU for 1 hour, pre-treated or not with 10µM of ATRi (VE-821). WCE were immunoblotted with the indicated antibodies.

Source data are provided as a Source Data file.

Supplementary Figure 6

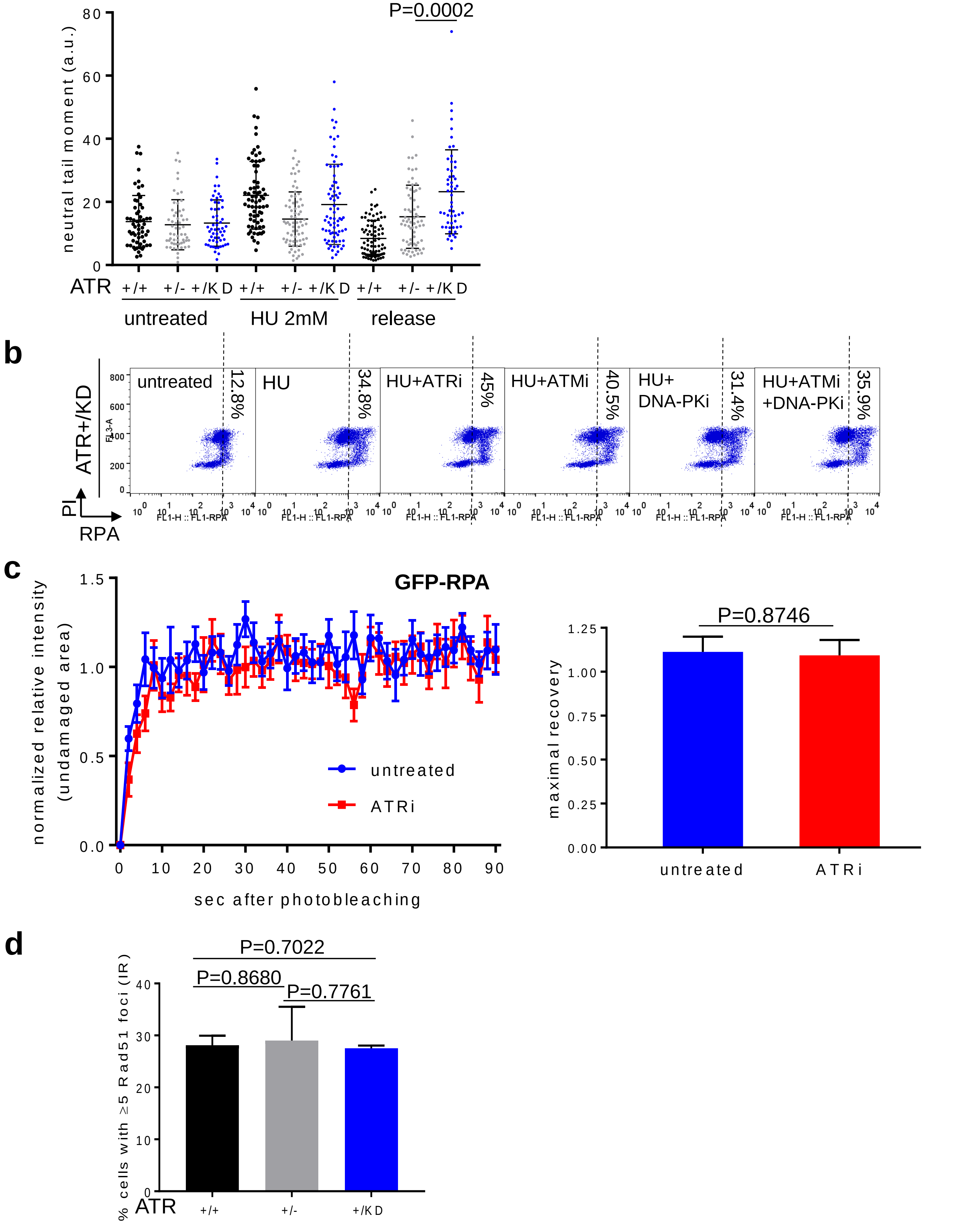

Supplementary Figure 6 Increased DNA breaks and chromatin bound RPA in *Atr*$^{+/KD}$ MEFs. (**a**) Dot plot of neutral comet tail moments of *Atr*$^{+/+}$, *Atr*$^{+/-}$ and *Atr*$^{+/KD}$ cells left untreated, treated with 2mM HU for 3 hours or released from HU in unchallenged medium for an additional 3 hours. The mean values ± SD are shown and unpaired two-tailed t test was used. (**b**) The chromatin-bound RPA fraction in *Atr*$^{+/KD}$ MEFs treated with 0.2 mM HU with or without the indicated checkpoint inhibitor is shown. ATRi (VE-821) and CHK1i (LY603218) were used at 10 µM final concentration, ATMi (KU-55933) at 15 µM and DNA-PKcsi (NU7441) at 5 µM. The dotted lines were arbitrarily set at 10^3 and mark the separation between the RPA chromatin-bound positive ($>10^3$) and negative ($<10^3$) cells. The percentages of RPA positive cells are reported for each treatment. (**c**) Quantification of the recovery of GFP-RPA relative intensity in an undamaged area after bleaching, in untreated or ATRi (VE-821) treated U2OS cells. The means ± SEM of the maximal recovery of GFP-RPA are reported and unpaired two-tailed t test was used for the statistical analysis. (**d**) *Atr*$^{+/+}$, *Atr*$^{+/-}$ and *Atr*$^{+/KD}$ MEFs were treated with IR (5 Gy) and let recover for 10 hours. Nuclei were stained for Rad51 and DAPI. Data are representative of two independent experiments and a total of about 1,000 cells were analyzed for every genotype. The mean values ± SD are shown and unpaired two-tailed t test was used for the statistical analysis.

Source data are provided as a Source Data file.

Supplementary Figure 7

Figure 4e uncropped

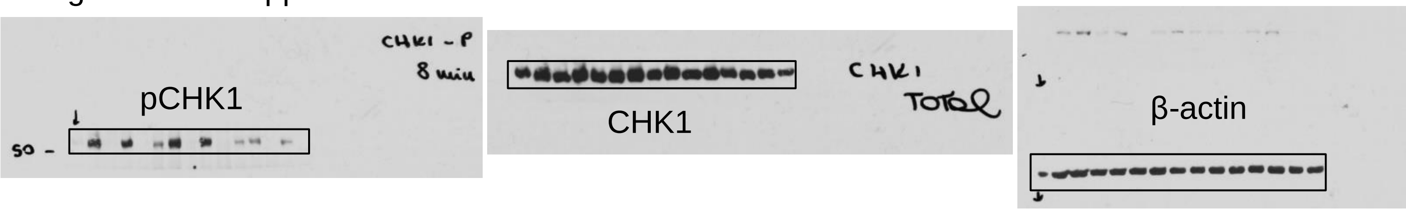

Figure 4f uncropped

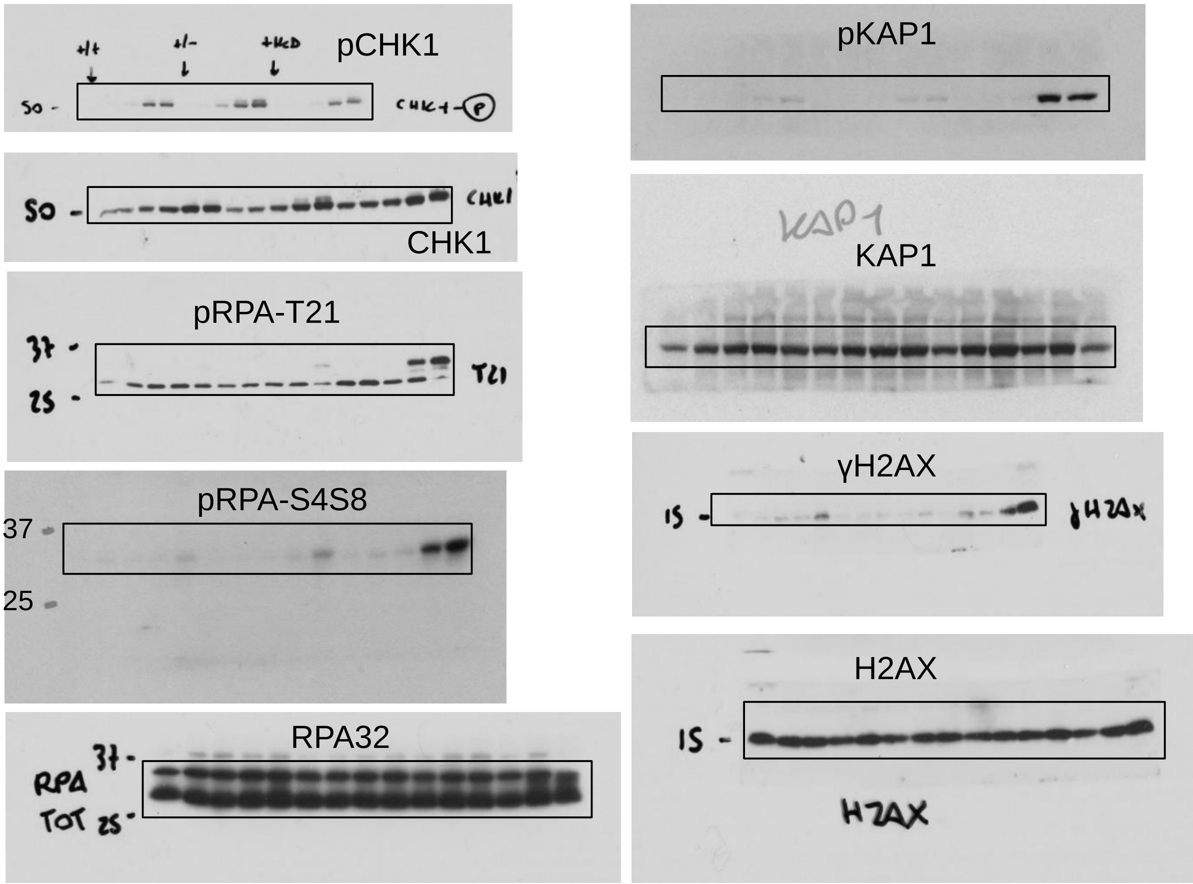

Figure 4g uncropped

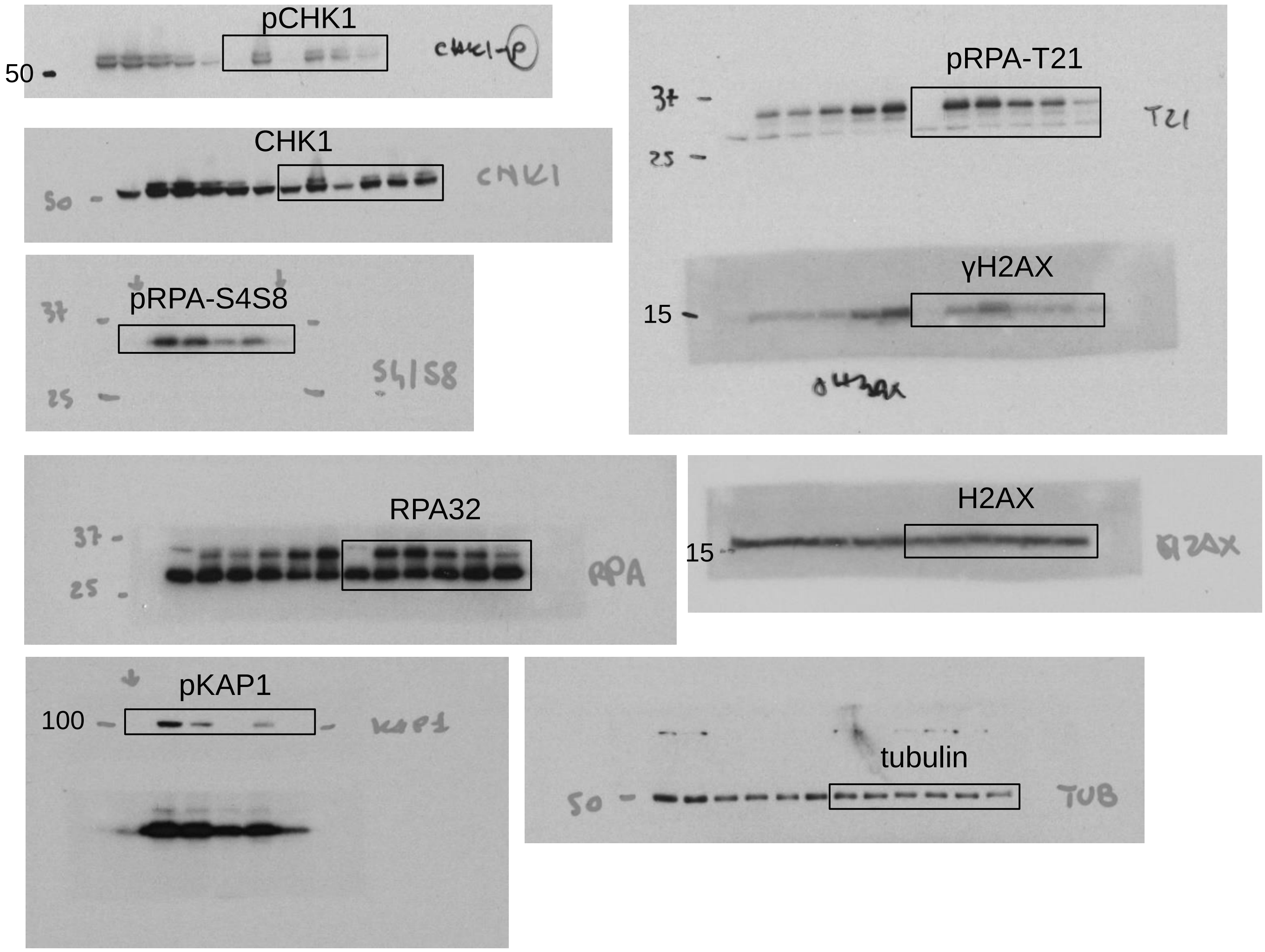

Figure 5d uncropped

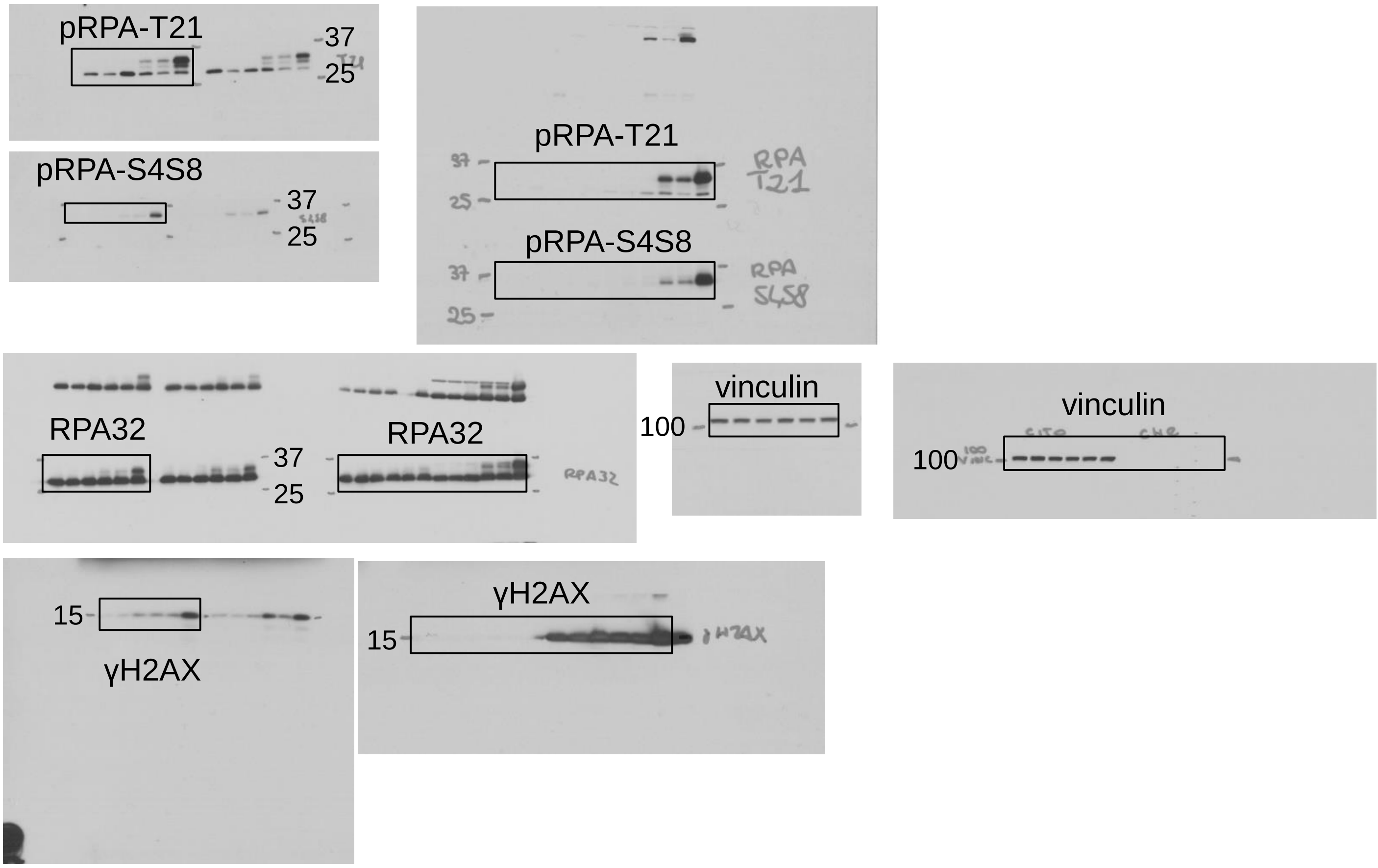

Figure 6a uncropped

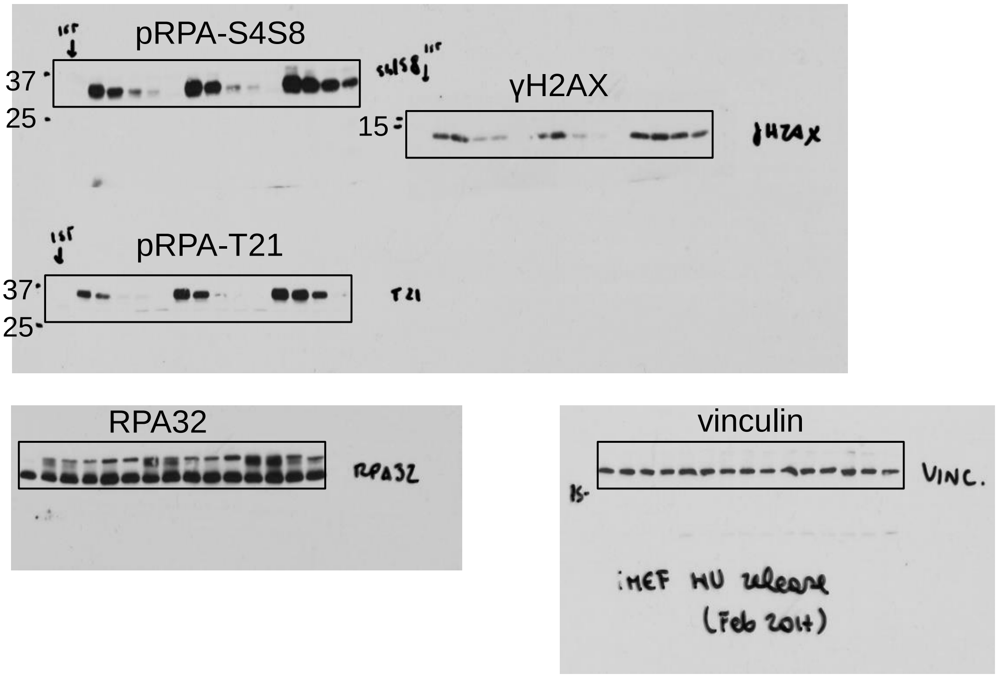

Supplementary Figure 7 Uncropped images of all the western blots shown in the main Figures.
