# Supplementary material for: Kinase-dead ATR differs from ATR loss by limiting the dynamic exchange of ATR and RPA
Source: Nat Commun. 2018 Dec 17;9:5351. doi: 10.1038/s41467-018-07798-3 (PMC6297235; doi:10.1038/s41467-018-07798-3)
Supplement: Supplementary file 3 — Reporting Summary [file 41467_2018_7798_MOESM3_ESM.pdf]

nature research

Corresponding author(s): Dr Shan Zha

Reporting Summary

Nature Research wishes to improve the reproducibility of the work that we publish. This form provides structure for consistency and transparency in reporting. For further information on Nature Research policies, see Authors & Referees and the Editorial Policy Checklist.

Statistical parameters

When statistical analyses are reported, confirm that the following items are present in the relevant location (e.g. figure legend, table legend, main text, or Methods section).

n/a	Confirmed	
☐	☒	The exact sample size (*n*) for each experimental group/condition, given as a discrete number and unit of measurement
☐	☒	An indication of whether measurements were taken from distinct samples or whether the same sample was measured repeatedly
☐	☒	The statistical test(s) used AND whether they are one- or two-sided<br>*Only common tests should be described solely by name; describe more complex techniques in the Methods section.*
☒	☐	A description of all covariates tested
☒	☐	A description of any assumptions or corrections, such as tests of normality and adjustment for multiple comparisons
☐	☒	A full description of the statistics including central tendency (e.g. means) or other basic estimates (e.g. regression coefficient) AND variation (e.g. standard deviation) or associated estimates of uncertainty (e.g. confidence intervals)
☐	☒	For null hypothesis testing, the test statistic (e.g. *F*, *t*, *r*) with confidence intervals, effect sizes, degrees of freedom and *P* value noted<br>*Give P values as exact values whenever suitable.*
☒	☐	For Bayesian analysis, information on the choice of priors and Markov chain Monte Carlo settings
☒	☐	For hierarchical and complex designs, identification of the appropriate level for tests and full reporting of outcomes
☒	☐	Estimates of effect sizes (e.g. Cohen's *d*, Pearson's *r*), indicating how they were calculated
☐	☒	Clearly defined error bars<br>*State explicitly what error bars represent (e.g. SD, SE, CI)*

Our web collection on statistics for biologists may be useful.

Software and code

Policy information about availability of computer code

Data collection	CellQuest Pro on FACScalibur (Becton Dickinson)<br>Metafer and Metacyte scanning imaging platforms (MetaSystems) on Carl Zeiss AxioImager Z2 microscope<br>NIS-Elements AR software on Nikon 80i fluorescence microscope (Nikon, Inc)<br>NIS Element High Content Analysis software (Nikon, Inc) on a Nikon Ti Eclipse inverted microscope (Nikon, Inc) equipped with A1 RMP (Nikon, Inc) confocal microscope system (Nikon, Inc) and Lu-N3 Laser Units (Nikon, Inc)
Data analysis	GraphPad Prism 7<br>Microsoft Excel 2016<br>FlowJo X<br>Isis fluorescence imaging platform (MataSystems)<br>CometScore version 1.5<br>ImageJ (Image processing and analysis in Java)<br>Fiji

For manuscripts utilizing custom algorithms or software that are central to the research but not yet described in published literature, software must be made available to editors/reviewers upon request. We strongly encourage code deposition in a community repository (e.g. GitHub). See the Nature Research guidelines for submitting code & software for further information.

Data

Policy information about availability of data

All manuscripts must include a data availability statement. This statement should provide the following information, where applicable:

- Accession codes, unique identifiers, or web links for publicly available datasets
- A list of figures that have associated raw data
- A description of any restrictions on data availability

The authors declare that data supporting the findings of this study are available with the paper and its supplementary information files. The source data underlying Figs 1b, c, e, f, 2a-e, 3b-c, 4a, b, e-h, 5a, c, d, 6 a-d and Supplementary Figs 1a, c, d, g, h, 2c-f, 3c-d, 4 a, c-d, 5a, c ,e-g, 6a, c-d are provided as a Source Data file.

Field-specific reporting

Please select the best fit for your research. If you are not sure, read the appropriate sections before making your selection.

☒ Life sciences ☐ Behavioural & social sciences ☐ Ecological, evolutionary & environmental sciences

For a reference copy of the document with all sections, see nature.com/authors/policies/ReportingSummary-flat.pdf

Life sciences study design

All studies must disclose on these points even when the disclosure is negative.

Sample size	Sample size was determined according to common standards in the field. Several (n>=3) independent mice and independent primary cell line (B cells or MEFs) of each genotype were analyzed for each phenotype described.
Data exclusions	All valid experimental data were included in the analyses.
Replication	All experiments presented here were validated in independent experiments and/or with independently derived cell lines or animals.
Randomization	Applied when possible.
Blinding	Applied when possible.

Reporting for specific materials, systems and methods

Materials & experimental systems

n/a	Involved in the study	
☒	☐	Unique biological materials
☐	☒	Antibodies
☐	☒	Eukaryotic cell lines
☒	☐	Palaeontology
☐	☒	Animals and other organisms
☒	☐	Human research participants

Methods

n/a	Involved in the study	
☒	☐	ChIP-seq
☐	☒	Flow cytometry
☒	☐	MRI-based neuroimaging

Antibodies

Antibodies used

Western Blot:
rabbit polyclonal pKAP1 (S824), A300-767A, Bethyl Laboratories
rabbit monoclonal KAP1 (TIF1β), C42G12, Cell Signaling, #4124
rabbit monoclonal pCHK1 Ser245 (133D3), Cell Signaling, #2348
mouse monoclonal CHK1 (2G1D5), Cell Signaling, #2360
rabbit monoclonal anti-RPA32/RPA2 (phospho T21), Abcam, ab109394
rabbit polyclonal phospho RPA32 (S4/S8), A300-245A, Bethyl Laboratories
rabbit polyclonal RPA32 antibody, A300-244A, Bethyl Laboratories
rabbit polyclonal anti phospho H2AX (Ser139), 07-164, Millipore
rabbit polyclonal anti-histone H2AX, 07-627, Millip2ore
rabbit polyclonal ATR antibody, Cell Signaling, #2790
mouse monoclonal anti-vinculin antibody, clone V284, 05-386, Millipore

mouse monoclonal anti-β-actin antibody, clone AC-15, A1978, Sigma
mouse monoclonal anti α-tubulin (DM1A), CP06, Millipore

Immunofluorescence:
rabbit polyclonal anti-Rad51 (Ab-1), PC-130, Calbiochem
rabbit polyclonal anti-SCP3, Abcam, ab15093
mouse monoclonal anti-SCP3 [Cor 10G11/7], Abcam, ab97672
mouse anti-human MLH1, clone G168-15, BD Pharmingen, cat. No. 550838
rat monoclonal anti-BrdU antibody [BU1/75 (ICR1)], Abcam, ab6326
mouse anti-BrdU, clone B44, Beckton Dickinson, cat. No. 347580
anti-DNA antibody, single-stranded, clone 16-19, MAB3034, Millipore

FACS analysis:
FITC-conjugated mouse anti-BrdU antibody, BD Pharmingen, cat. No. 556028
mouse monoclonal anti-Replication Protein A (Ab-3), RPA34-20, NA19L, Millipore
FITC rat anti-mouse IgG1, clone A85-1, BD Pharmingen, cat. No. 553443
PE-Cyanine5 rat anti-mouse CD45R/B220, clone RA3-6B2, BD Pharmingen, cat. No. 553091
FITC rat anti-Cd11b, clone M1/70, BD Pharmingen, cat. No. 553310
APC anti-mouse Ly-6G/Ly-6C (Gr-1), clone RB6-8C5, BioLegend
Goat anti-mouse IgM, Human ads-PE, SouthernBiotech, cat. No. 1020-09
PE rat anti-mouse CD4, clone GK1.5, BD Pharmingen, cat. No. 553730
FITC anti-mouse CD8a, clone 53-6.7, BioLegend
PE-Cyanine 5 CD3e monoclonal antibody, 145-2C11, Invitrogen, 15-0031-63

Validation

All antibodies described and used in this study have been extensively validated by the manufacturers (see manufacturer web site for details) and in our control experiments (including negative control as well as wild type animals that show expected distribution of immune cell populations).

Eukaryotic cell lines

Policy information about cell lines

Cell line source(s)

Primary and immortalized MEFs (mouse embryonic fibroblasts), B cells and U2OS

Authentication

The primary and immortalized MEFs and B cells were genotyped to ensure their identity. U2OS with stably expression of GFP-ATR was validated based on GFP expression. The results were independent validated via transient transfection of GFP/RFP-ATR or ATR-KD in independently maintained U2OS cell line (from ATCC HTB-96).

Mycoplasma contamination

Mycoplasma is a lethal threat to primary cell culture (e.g. primary B cells and MEFs). Thus we test mycroplasma regularly with sensitive qPCR methods that detects a conserved 16s rDNA presents in most laboratory mycoplasma species. This is the same method that has been used by commercial test kits (e.g. Sigma and MD Biosciences).

Commonly misidentified lines
(See ICLAC register)

Not used

Animals and other organisms

Policy information about studies involving animals; ARRIVE guidelines recommended for reporting animal research

Laboratory animals

All the mice used in the main and supplementary figures are of pure 129/Sv strain. ATR null and ATR conditional mice were generously provided by Dr. Eric Brown (UPENN), and are also in the 129/Sv background. Both female and male mice were used for lymphocyte analyses (4-12 weeks). Only male mice were used for spermatogenesis studies (age 3 week to 26 weeks). A previously characterized ROSA26(CRE-ERT2) allele (J Clin Invest. 115:3484-93) was used to generate Rosa26+/Cre-ERT2 ATRC/-(C/KD) MEFs presented in supplementary figure 1c and 1d.

Wild animals

N/A

Field-collected samples

N/A

Flow Cytometry

Plots

Confirm that:

☒ The axis labels state the marker and fluorochrome used (e.g. CD4-FITC).

☒ The axis scales are clearly visible. Include numbers along axes only for bottom left plot of group (a 'group' is an analysis of identical markers).

☒ All plots are contour plots with outliers or pseudocolor plots.

☒ A numerical value for number of cells or percentage (with statistics) is provided.

Methodology

Sample preparation	Sample preparation is extensively described in Methods section. For lymphocyte development studies, mice cells are recovered from spleen, thymus or bone marrow and are resuspended in PBS 1X and kept on ice before staining with indicated antibodies. For Class Switch Recombination (CSR), splenic CD43- B cells are purified using MACS magnetic purification system and Myltenyi Biotec columns. B cells are cultured in RPMI supplemented with IL-4 and anti-CD40 and stained with IgG1 and B220 fluorescent antibodies every day after stimulation until Day 4.5. For cell cycle analysis, mouse embryonic fibroblasts are trypsinized, washed in PBS 1X, fixed in EtOH 70% at 4C. Samples are processed as indicated in the protocol for BrdU and PI staining described in Methods section.
Instrument	FACScalibur flow cytometer (BD Biosciences).
Software	CellQuest Pro software for data collection on FACScalibur. FlowJo X software for data analysis.
Cell population abundance	For development analyses, primary and secondary lymphoid tissues were directly isolated for FACS analyses without purifications. For CSR analyses, splenic B cells were purified to exclude CD43+ (most T cells and myeloid cells) cells. The purified splenic B cell population were routinely analyzed to ensure > 80% B220+IgM+ naive B cells.
Gating strategy	The gating strategy is based on the live cell population and on the marker that has to be shown. A figure exemplifying the strategy is reported in the main or in the supplementary figures for every flow cytometry experiment performed.

☒ Tick this box to confirm that a figure exemplifying the gating strategy is provided in the Supplementary Information.
